# Analyzing Risk Communication, Trust, Risk Perception, Negative Emotions, and Behavioral Coping Strategies During the COVID-19 Pandemic in China Using a Structural Equation Model

**DOI:** 10.3389/fpubh.2022.843787

**Published:** 2022-05-30

**Authors:** Junwang Gu, Rong He, Xuanhui Wu, Jing Tao, Wenhui Ye, Chunmei Wu

**Affiliations:** School of Public Health and Health Management, Gannan Medical University, Ganzhou, China

**Keywords:** emergency risk management, influencing factors, risk perception, negative emotions, behavioral coping strategies, structural equation model

## Abstract

**Objective:**

Risk communication and the degree of trust are major factors that affect the public's behavioral coping strategies and play an important role in emergency risk management. However, the internal formation mechanism involved in the public's psychological behavior remains unclear. This study aimed to investigate the association among risk communication, trust, risk perception, negative emotions, and behavioral coping strategies during the coronavirus disease 2019 (COVID-19) pandemic, and to identify and quantify the factors that influence public behavior.

**Methods:**

We launched an online survey through social media from April to July 2020 in China. Relevant data were elicited using a self-designed questionnaire that mainly examined respondent characteristics, risk communication, trust, risk perception, negative emotions, protective coping behavior, and excessive coping behavior in the context of the COVID-19 pandemic. A total of 735 valid responses were obtained. A structural equation model was then used to explore relationship pathways among the components.

**Results:**

The higher the degree of risk communication (β = −0.10, *p* < 0.05) and trust (β = −0.22, *p* < 0.001), the lower the public risk perception. Risk communication and trust had a direct effect on public behavioral coping strategies during the COVID-19 pandemic. The higher the level of risk communication (β = 0.14, *p* < 0.001) or trust (β = 0.48, *p* < 0.001), the more likely it was that this would encourage the public to adopt protective coping behaviors, while the public was less likely to engage in excessive coping behaviors as the degree of trust increased (β = −0.12, *p* < 0.01). Risk perception influenced by poor risk communication and trust generated negative emotions (β = 0.31, *p* < 0.001), and such negative emotions further positively influenced public behavioral coping strategies (whether protective [β = 0.09, *p* < 0.05] or excessive [β = 0.24, *p* < 0.001] behaviors).

**Conclusion:**

Risk communication, trust, risk perception, and negative emotions were significantly directly or indirectly related to public behavior. The findings provide useful information for emergency risk management and a theoretical basis for follow-up research on public coping behavior during the COVID-19 pandemic.

## Introduction

Coronavirus disease 2019 (COVID-19), a fulminant infectious disease, has been sweeping the globe, causing extensive losses of life and generating significant worldwide concern (1). The COVID-19 pandemic has exposed global weaknesses in infection control practices, especially in the operational efficiency of health emergency services (2). Thus, there is an urgent need to establish a higher-level emergency management system when facing new challenges during such demanding public health emergencies.

Public risk perception (RP) changes with time, which is an important consideration in public health emergencies and risk management decision-making (3), while its role can be easily overlooked in the construction of emergency management systems. RP is defined as the subjective judgment people make about the characteristics and severity of risks (4), which was likely to be influenced by many factors like personal characteristics, health literacy during a crisis such as the COVID-19 pandemic (5–7).

The COVID-19 pandemic is deeply affecting all aspects of societies, this continuous long-term battle with the pandemic not only poses a huge burden on healthcare but also affects our daily life, social interactions, and work performance (8). COVID-19 information rapidly changes, in response, WHO developed risk communication (RC) and community engagement to help support efforts to promote uniform messaging for COVID-19 crisis (9). RC, according to a WHO guideline, is “the real-time exchange of information, advice and opinions between experts, community leaders, officials and the people who are at risk and is an integral part of any emergency response” (10). Therefore, from a public health perspective, RC is a critical factor for emergency management. It has been reported that RC may have direct and indirect positive effects on protective behaviors (11). Furthermore, RP mediates the relationship between RC and protective behaviors (11). A qualitative study revealed that when public RP was influenced by relevant information, the public's protective behavior was subsequently modified (12). The massively developing social network phenomenon has become the main dissemination platform for COVID-19 information and has attracted significant attention from emergency management agencies. One survey showed that the frequency of media use was positively associated with higher RP (13). Social media has become an important channel by which public could seek and share a staggering amount of health-related information and more so during the outbreak of COVID-19 (14). Social media exposure to COVID-19 information influences the adoption of preventive attitudes and behaviors by shaping RP (15). Informational support from the media plays one of the most critical role in facilitating individuals' adoption of compliance with anti-pandemic behavior (16). While there has been increasing attention to the role of social media during Covid-19 crisis, little is known about how social media can affect RP and public behaviors.

Trust is a key factor that encourages people to comply with public health regulations. One online survey indicated that higher trust in governmental organizations was linked to greater compliance in terms of adopting protective behaviors during the second wave of the Covid-19 outbreak (17). People with high social trust perceive more risks than do those with low social trust (18). Other factors, such as knowledge (6) and gender (7) are also important influencing factors in shaping public RP.

Currently, as part of an overall consideration of risk control, governments from different nations facing the spread of the global COVID-19 pandemic are imposing strict and severe mitigation measures to influence people's behaviors (19, 20). RP has been a major factor affecting people's behavioral coping strategies during the COVID-19 pandemic and has had the greatest impact on adherence to preventive measures (21–23). The perception of health risks plays a key role in the response to health emergencies, affecting risk management (4) and individual behaviors (24, 25). Based on prior knowledge or social observation, it could be hypothesized that high-level RP would cause the public to overreact to the crisis, further producing irrational and excessive behaviors, such as hoarding (26), panic buying (27), and a bandwagon effect led by rumors (herding behavior) (28, 29); while low-level RP would not be conducive to the public adopting positive self-protection behaviors, such as face-mask usage (30), social distancing (31, 32), and appropriate hygiene measures (33), leading to an aggravated risk of epidemic spread. Such a hypothesis requires investigation, which we undertook in this study.

The COVID-19 outbreak has brought increasingly psychological distress to the worldwide population (34, 35). Some scholars (35–37) confirmed the results and underlying mechanism of studies on the Italian population who reported high concomitance of anxiety symptomatology and sleep disorders during the COVID-19 pandemic, with an increased risk of post-traumatic stress disorder symptoms (PTSD) occurrence. Determining the susceptibility factors affecting the emotional response to COVID-19 is of critical importance for improving psychologically based epidemic-crisis interventions. Research has shown that RP is positively related to depressive states (38), and a higher RP concerning COVID-19 is notably associated with less positive or more negative emotions (NE) (39). NE, such as worry, fear, and anxiety, have been consistently associated with RP and could be significant indicators of RP (40). Studies have shown that people who perceive more risks also report higher levels of anxiety (41). Public RP increases the public's state of anxiety and then increases their willingness to panic buy (29). The above research suggests that RP may have a direct effect on public emotion during the current pandemic, and that COVID-19-related NE may be a key explanatory factor linking public behavioral coping strategies and RP.

**Figure 1:**
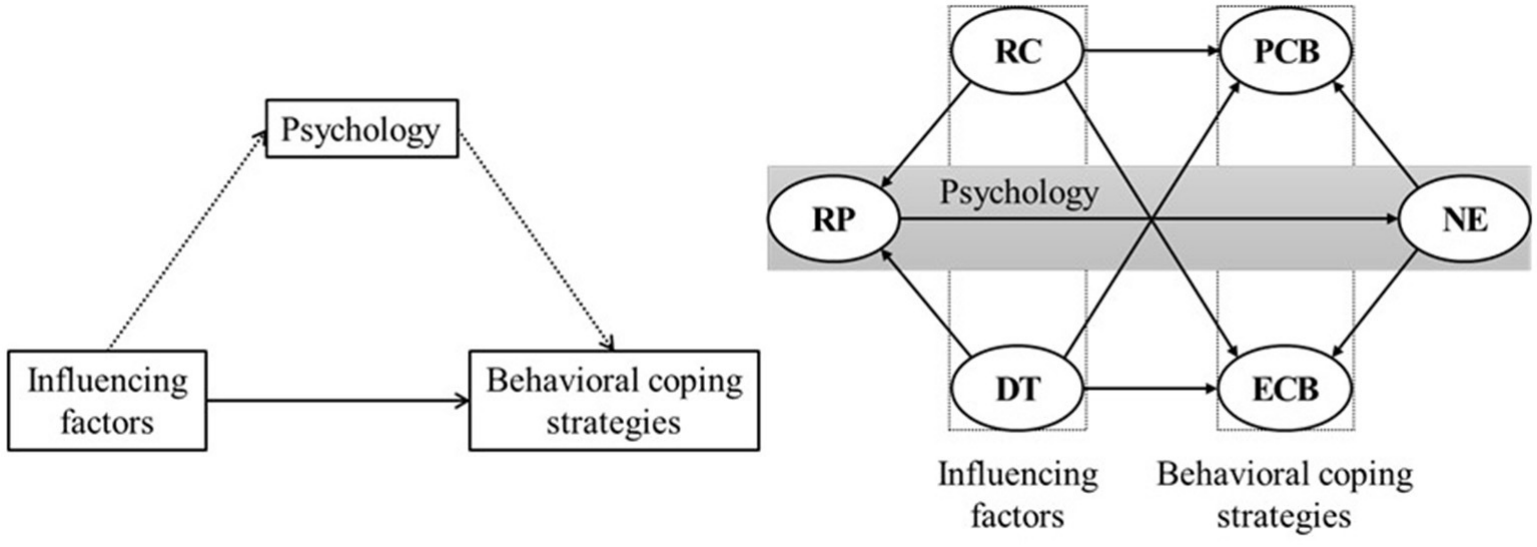
Structural equation model hypothesis. RC, Risk communication; DT, degree of trust; RP, risk perception; NE, negative emotions; PCB, protective coping behavior; ECB, excessive coping behavior.

Over recent decades, many behavioral risk management studies have focused on external influencing factors, such as RC, trust, and personal characteristics, but have not elucidated the internal mechanisms involved. Therefore, to further explore the internal formation mechanism involved in the public's psychological behavior during the COVID-19 pandemic, a relationship model comprising influencing factors such as public RP, NE, and behavioral coping strategies was constructed using a structural equation model (SEM) (Figure 1).

Accordingly, we will assess the relationships among influencing factors such as RC, trust, public RP, NE, and behavioral coping strategies (i.e., protective coping behavior [PCB], excessive coping behavior [ECB]); the mediating effect of NE on RP and behavioral coping strategies; and the effects of these influencing factors on public behavioral coping strategies when faced with COVID-19 from a psychological perspective (11, 17, 18, 21, 39). Our study is intended to provide a theoretical basis for better targeted epidemic-crisis interventions.

## Materials and Methods

### Respondents

We launched an online survey through social media, including Tencent WeChat or QQ and Sina Weibo, that was available from April to July 2020 in China. A total of 781 questionnaires were distributed, and incomplete or erroneously completed questionnaires were excluded. Finally, 735 valid responses were obtained with an efficiency recovery rate of 94%.

### Questionnaire

Data were elicited using a self-designed questionnaire in line with the relevant literature. The questionnaire consisted of three parts: (1) basic personal information of the respondents who participated in the survey, (2) each measurement scale, and (3) other items such as risk communication mediums. After the questionnaire was piloted, some questions were excluded.

The study protocol was approved by the ethics review board of Gannan Medical University, and the study was conducted in accordance with the ethical guidelines of the Declaration of Helsinki and relevant policies in China. Informed consent forms obtained from the participants stated that the questionnaire was to be completed anonymously and that the information provided was confidential.

### Measurement

#### Risk Communication (RC)

RC, as a key link in the entire process of emergency response, is defined as the exchange of real-time information, advice, and opinions between experts and people facing threats to their health, economic, or social well-being (10). Social media has become the main method to ensure appropriate RC and plays a critical role in sharing and transmitting risk information in time and effectively (42). In this study, we used a self-designed scale based on the relevant literature (26), namely, a 5-point Likert scale, where 1 corresponded to “*totally disagree*” and 5 to “*totally agree*” for the following three questions: (RC01) “Do you think media reports affected your judgment of the COVID-19 pandemic?;” (RC02) “Do you believe media reports on COVID-19 are true?;” and (RC03) “Do you believe that government departments are timely and transparent in the release of pandemic information?”

#### Degree of Trust (DT)

Trust involves an overall positive expectation concerning the worthiness of words, promises, and statements of either another person (interpersonal trust) or an institution (social trust). Based on previous literature (43, 44), we considered that COVID-19-related trust could be effectively measured in terms of beliefs related to “competency – having technical proficiency.” Specifically, in our study, the degree of trust (DT) was measured as the perceived or expected trustworthiness of others (e.g., government, medical workers), which was assessed using a 5-point Likert scale where one corresponded to “*very distrusting*” and 5 to “*very trusting*” for the following 4 questions: (DT01) “Can the pandemic be effectively controlled?;” (DT02) “What is your DT in the pandemic control ability of medical workers?;” (DT03) “What is your DT in your self-protection capabilities?;” and (DT04) “What is your DT in the government's ability to prevent and control the pandemic?”

#### Risk Perception (RP)

Owing to different risk scenarios and research methods, the RP dimension has not been consistently defined; however, the controllability of risk is the most representative dimension employed in different fields, for example, the field of natural disasters and food safety (45). Slovic (4) claimed that people's RP of crisis events can be measured from the two dimensions of familiarity and controllability. In this study, we chose controllability as the RP measurement index, with five aspects of risk events investigated, namely, etiology, transmission, cure, preventive measures, and prognosis. RP was assessed using a 5-point Likert scale for the following five questions: “In terms of the controllability of the COVID-19 pandemic, what is your RP concerning its etiology (RP01), transmission (RP02), cure (RP03), preventive measures (RP04), and prognosis (RP05)?” (Responses ranging from: 1 “*totally controllable*” to 5 “*totally uncontrollable*”).

#### Negative Emotions (NE)

NE were assessed using a 5-point Likert scale (ranging from 1 “*never*” to 5 “*always*”) in relation to the following questions: “What was your worry (NE01), fear (NE02), and anxiety (NE03) frequency during the COVID-19 pandemic?”

#### Protective Coping Behavior (PCB)

PCB was measured using a 5-point Likert scale (ranging from 1 “*never*” to 5 “*always*”) in relation to the following questions: (PCB01) “Did you reduce contact with others during the pandemic?;” (PCB02) “Did you always wear a mask when you went out during the pandemic?;” (PCB03) “Did you try to avoid going to crowded places for activities during the pandemic?;” (PCB04) “Did you increase the amount of handwashing during the pandemic?;” (PCB05) “Did you open windows more often for ventilation during the pandemic?;” (PCB06) “Did you reduce the number of dinner parties held during the pandemic?;” (PCB07) “Did you increase exercise during the pandemic?;” (PCB08) “Were you more proactive in paying attention to and seeking health information during the pandemic?;” (PCB09) “Did you remind your family or friends to take measures to prevent and treat infection due to COVID-19 during the pandemic?;” and (PCB10) “Did you try to avoid contact with wild animals during the epidemic?”

#### Excessive Coping Behavior (ECB)

Based on social observations, ECB was assessed using three multiple choice questions, as follows: (ECB01) “Did you panic buy during the pandemic? If yes, please indicate whether any of the following items were involved: personal protective equipment, disinfectant, antiviral drugs, other directly pandemic-related purchases, or none of these;” (ECB02) “Did you hoard during the pandemic? If yes, please indicate whether any of the following items were involved: personal protective equipment, disinfectant, antiviral drugs, other directly pandemic-related purchases, or none of these;” (ECB03) “Were you influenced by a bandwagon effect led by rumor (herding behavior)? If yes, please indicate whether any of the following was involved: taking Shuanghuanglian (a drug with no preventive effect) to prevent COVID-19, sterilizing with white vinegar, taking other antiviral drugs while not been infected, other similar behavior, or none of these.” One point was assigned for each type of behavior, with points ranging from 0–4 points for each item.

### Statistics

For statistical analysis, descriptive measures were performed using IBM SPSS (version 20.0, USA) software to summarize the principal results. Confirmatory factor analysis (CFA), assessment of normality, and SEM analyses were performed using SPSS AMOS (version 17.0, IBM Analytics, USA) software. Statistical significance was set at *p* < 0.05.

Compared with other methods [e.g., traditional multivariate statistical analysis (46), artificial neural network model (47)], SEM can construct hypothetical paths before data analysis, further perform CFA, and analyze the structural relationship between potential variables. This sample size of 735 was considered adequate for our SEM analyses, as the minimum satisfactory sample size recommended for performing a SEM procedure is 200 participants (48). For questionnaire validity and reliability, CFA testing, the index of composite reliability (CR) and average variance extracted (AVE), and Cronbach's alpha (α) were employed. The χ^2^/df, confirmatory factor index (CFI), expected cross validation index (ECVI), incremental fit index (IFI), normed fit index (NFI), parsimony comparative fit index (PCFI), root mean squared error of approximation (RMSEA) were adopted to determine whether the models fit the data (the evaluation Standard were showed in **Table 4**).

## Results

### Respondent Characteristics

The characteristics of the respondents are set out in Table 1. A total of 341 (46.4%) male participants and 394 (53.6%) female participants, mainly aged 18–24 years (72.5%), were included in this survey. The educational background of the respondents was mainly bachelor's degree or above (60.3%). Approximately 39.3% of the respondents had a medical background, and 61.9% of the respondents were students. Of the respondents, 30.1% had an annual income of <30,000 Yuan, and 39.5% had an income of 30,000–80,000 Yuan. Of the participants, 86.3% lived in rural areas during the COVID-19 pandemic, while the rest (13.7%) lived in urban areas.

**Table 1:** Characteristics of respondents.

**Variable**	**Category**	**Frequency**	**Percentage (%)**
Gender	Male	341	46.4
	Female	394	53.6
Age (years)	≤17	12	1.6
	18–24	533	72.5
	25–34	83	11.3
	35–44	46	6.3
	≥45	61	8.3
Educational level	Junior high or below	73	9.9
	Senior high or technical secondary school	95	12.9
	College degree	124	16.9
	Bachelor or above degree	443	60.3
Medical related major?	Yes	236	39.3
	No	364	60.7
Profession	Student	455	61.9
	Medical staff	27	3.7
	Teacher	28	3.8
	Civil servants	7	1.0
	Farmer	18	2.4
	Worker	28	3.8
	Others	172	29.5
Income (Yuan)	<30,000	221	30.1
	≥30,000 and <80,000	290	39.5
	≥80,000 and <120,000	128	17.4
	≥120,000	73	9.9
Residence	Urban	101	13.7
	Rural town	634	86.3
Information or knowledge source^*^	Family, friends	271	36.9
	Health educators	318	43.3
	Official agencies	192	26.1
	Internet	590	80.3
	TV, newspapers	503	68.4
	Others	37	5.0
Information media^*^	Official media	520	70.7
	Search engine	376	51.2
	social network	510	69.4
	traditional media	410	55.8
	Others	60	8.2

* *MCQ, multiple-choice questions*.

A wide range of sources of information and knowledge about COVID-19 was identified, mainly comprising the Internet in terms of news websites and forums (80.3%), followed by television news and newspapers (68.4%), health educators in hospitals or communities (43.3%), family or friends (36.9%), official agencies such as health administration departments and Centers for Disease Control and Prevention (26.1%), and others (5.0%). COVID-19 pandemic information media comprised official media, such as government documents and press conferences (70.7%); social networks, for example, Sina Weibo, Tencent WeChat, or QQ (69.4%); traditional media such as radio, television, and newspapers (55.8%); search engines, such as Baidu and Google (51.2%); and others (8.2%).

### Confirmatory Factor Analysis (CFA)

We tested the affiliation between the measurement and latent variables using CFA. Some items were removed because of low loading values or cross loadings. Specifically, the items RC01, PCB07, PCB01, and ECB03 with standardized factor loadings <0.6 were deleted step-by-step. Items PCB03, PCB10, and PCB08 were deleted when a strong and significant correlation was found between sets of variables (PCB03 and PCB06, PCB10 and PCB06, PCB08, and PCB09) in terms of the latent variable PCB based on modification indices. The items in the final model are shown in Table 2, which includes 6 latent variables and 21 measurement variables.

**Table 2:** Structural validity and reliability.

**Measurement variable**	**Path**	**Latent variable**	**Standardized loading factor**	**Assessment of normality**				**CR**	**AVE**	**Cronbach's Alpha**
				**Skew**	**c.r**.	**Kurtosis**	**c.r**.			
RC02	←	RC	0.743	−0.473	−5.240	−0.316	−1.751	0.774	0.632	0.771
RC03	←	RC	0.844	−0.671	−7.429	0.345	1.907			
DT01	←	DT	0.896	−1.056	−11.686	1.297	7.180	0.904	0.703	0.900
DT02	←	DT	0.900	−1.165	−12.897	1.322	7.318			
DT03	←	DT	0.855	−1.512	−16.739	2.398	13.273			
DT04	←	DT	0.684	−0.524	−5.802	−0.214	−1.187			
RP01	←	RP	0.759	0.852	9.430	1.078	5.963	0.902	0.649	0.902
RP02	←	RP	0.811	0.941	10.418	1.231	6.811			
RP03	←	RP	0.829	0.917	10.152	1.288	7.129			
RP04	←	RP	0.852	1.211	13.408	1.887	10.445			
RP05	←	RP	0.773	0.779	8.619	0.954	5.278			
NE01	←	NE	0.729	0.027	0.295	−0.414	−2.291	0.870	0.692	0.867
NE02	←	NE	0.881	0.191	2.116	−0.253	−1.402			
NE03	←	NE	0.876	0.121	1.339	−0.447	−2.474			
PCB02	←	PCB	0.713	−1.624	−17.973	2.340	12.949	0.854	0.543	0.847
PCB04	←	PCB	0.808	−1.063	−11.764	0.595	3.291			
PCB05	←	PCB	0.809	−1.165	−12.896	1.263	6.989			
PCB06	←	PCB	0.587	−1.711	−18.935	2.446	13.538			
PCB09	←	PCB	0.743	−1.011	−11.185	0.490	2.713			
ECB01	←	ECB	0.750	0.914	10.114	0.109	0.601	0.807	0.677	0.800
ECB02	←	ECB	0.890	0.600	6.639	−0.429	−2.374			
Multivariate						170.370	74.305			

*c.r., critical ratio; CR, composite reliability; AVE, average variance extracted. RC, risk communication; DT, degree of trust; RP, risk perception; NE, negative emotions; PCB, protective coping behavior; ECB, excessive coping behavior*.

The model fit of χ2/df = 2.427 ≤ 3, RMSEA = 0.044 ≤ 0.05, estimated after CFA, showed that the scale had adequate structural validity. The AVE values of each latent variable were all >0.5 (49), with CR ≥0.7 (46): RC (α = 0.771, CR = 0.774, AVE = 0.632); DT (α = 0.900, CR = 0.904, AVE = 0.703); RP (α = 0.902, CR = 0.902, AVE = 0.649); NE (α = 0.867, CR = 0.870, AVE = 0.692); PCB (α = 0.847, CR = 0.854, AVE = 0.543); and ECB (α = 0.800, CR = 0.807, AVE = 0.677), indicating that each variable had good convergent validity and adequate reliability (α ≥ 0.7) (50). As shown in Table 3, the inter-factor correlation coefficients were ≤0.60, and the square roots of the AVE values were higher than the correlation coefficients, indicating that the discriminant validity of the factors was verified. The results of the assessment of normality (Table 2) showed that all measurement variables conformed to a normal distribution when the absolute value of skew and kurtosis were ≤ the corresponding critical ratio, in accordance with the requirements of the maximum likelihood method. Hence, we computed the SEM adopting maximum likelihood estimation.

**Table 3:** Discriminant validity.

**Factor (latent variable)**	**RC**	**DT**	**RP**	**NE**	**PCB**	**ECB**
RC	**0.632**					
DT	0.380	**0.703**				
RP	−0.181	−0.250	**0.649**			
NE	−0.005	−0.136	0.308	**0.692**		
PCB	0.325	0.524	−0.166	0.022	**0.543**	
ECB	0.012	−0.149	0.135	0.249	−0.021	**0.677**
Square root of AVE	0.795	0.838	0.806	0.832	0.737	0.822

*Values (bold) in diagonal are average variance extracted (AVE), and the off-diagonal values are inter-factor correlations. RC, risk communication; DT, degree of trust; RP, risk perception; NE, negative emotions; PCB, protective coping behavior; ECB, excessive coping behavior*.

### Structural Equation Model (SEM) Testing

**Figure 2:**
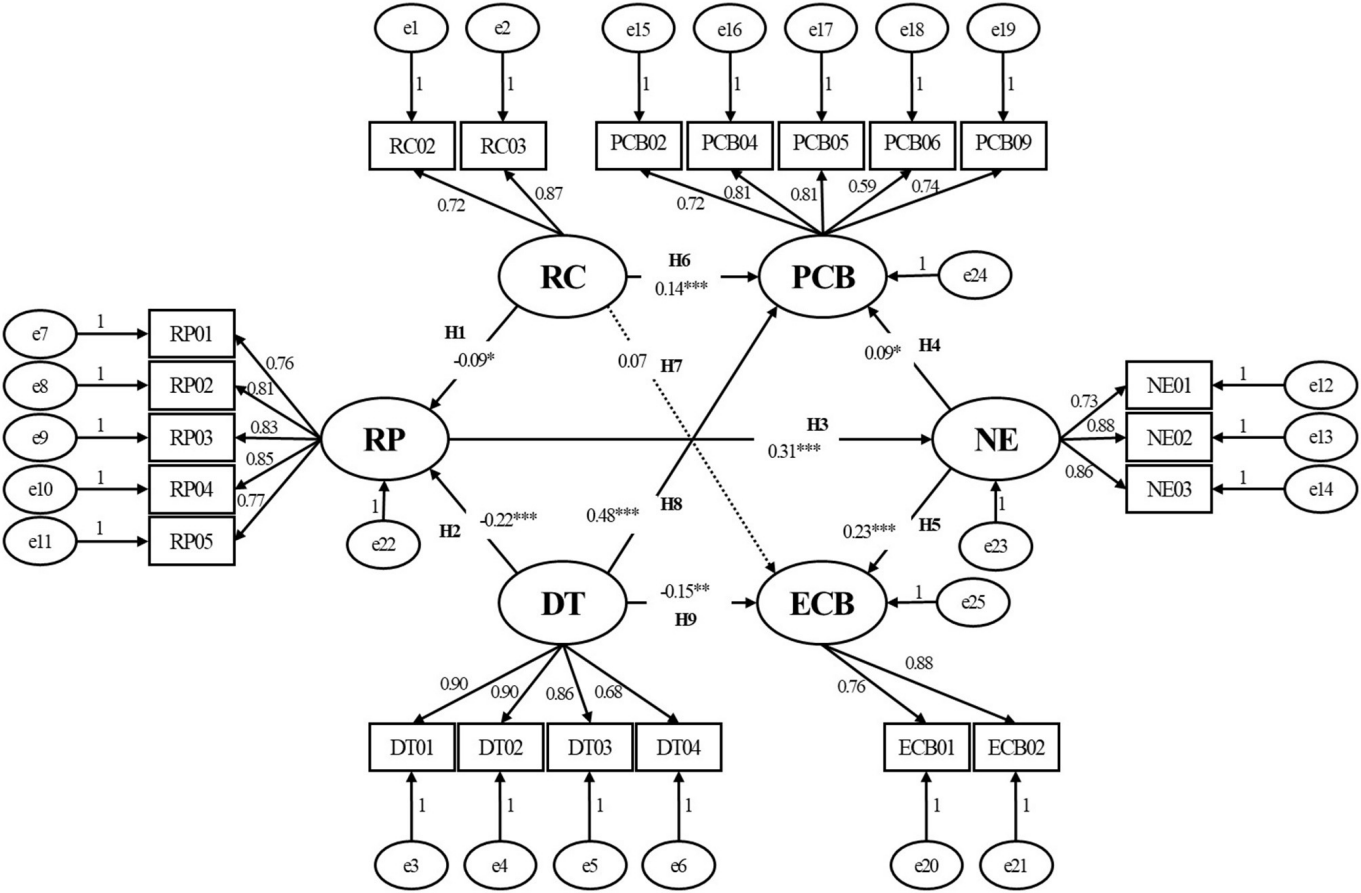
Model 1: Preliminary fit results of the theoretical model. *p < 0.05; **p < 0.01; ***p < 0.001. RC, risk communication; DT, degree of trust; RP, risk perception; NE, negative emotions; PCB, protective coping behavior; ECB, excessive coping behavior. The variables e1, e2,..., e25 represent the structure residuals.

**Figure 3:**
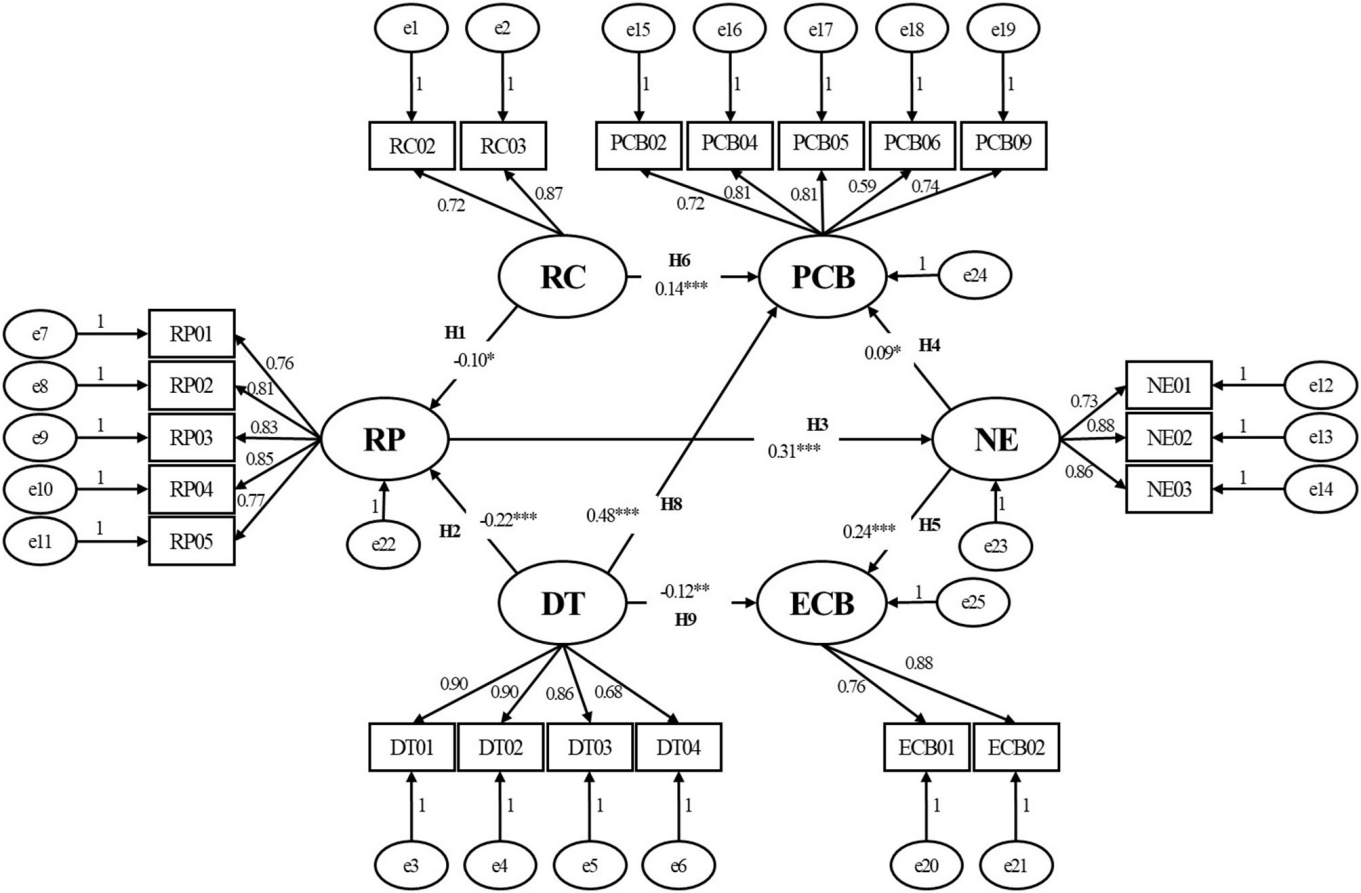
Model 2: The final structural equation model. *p < 0.05; **p < 0.01; ***p < 0.001. RC, risk communication; DT, degree of trust; RP, risk perception; NE, negative emotions; PCB, protective coping behavior; ECB, excessive coping behavior. The variables e1, e2,..., e25 represent the structure residuals.

To test our hypothesis, we constructed a structural equation model (Figure 2) with RC and DT as the exogenous variables, and PP, EM, PCB, and ECB as the endogenous latent variables (χ^2^/df = 2.408, RMSEA = 0.044). Path H7 (RC-ECB) was deleted because the path coefficient was significantly small (β = 0.07, *p* > 0.05). The final SEM is shown in Figure 3, with χ2/df = 2.406 and RMSEA = 0.044 (Table 4), indicating that the final model had an adequate fit for the observable data.

**Table 4:** Structural equation model fitting index.

**Model**	**χ^2^/df**	**NFI**	**IFI**	**CFI**	**RMSEA**
**After CFA**					
Model 0	2.427	0.950	0.970	0.970	0.044
Model 1	2.408	0.949	0.969	0.969	0.044
**Delete path H7 (*****β*** **=** **0.070**, ***p*** **=** **0.152)**					
Model 2	2.406	0.948	0.969	0.969	0.044
Evaluation standard	<5.000	>0.900	>0.900	>0.900	<0.050

*CFA, confirmatory factor analysis; CFI, confirmatory factor index; ECVI, expected cross validation index; IFI, incremental fit index; NFI, normed fit index; PCFI, parsimony comparative fit index; RMSEA, root mean squared error of approximation*.

After verifying and correcting the model, the paths were tested. Overall, our results (Table 5) were consistent with our hypothesis: (1) RC and DT were influencing factors of RP; the higher the degree of RC (β = −0.10, *p* < 0.05) or DT (β = −0.22, *p* < 0.001), the lower the public RP; (2) RC and DT had a direct effect on public behavioral coping strategies during the COVID-19 pandemic. The higher the level of RC (β = 0.14, *p* < 0.001) or DT (β = 0.48, *p* < 0.001), the more likely it was that the public were encouraged to adopt PCB, whereas the public were less likely to engage in ECB as DT increased during the COVID-19 pandemic (β = −0.12, *p* < 0.01). (3) RC and DT had an indirect effect on public behavioral coping strategies during the COVID-19 pandemic, and psychological factors may be an intermediary variable in relation to behavioral coping strategies stimulated by RC and DT. Specifically, RP as influenced by RC and DT could positively generate NE (β = 0.31, *p* < 0.001), which would further positively influence public behavioral coping strategies (whether protective [β = 0.09, *p* < 0.05] or excessive [β = 0.24, *p* < 0.001] behaviors). It was clear that NE had a stronger direct effect on ECB.

**Table 5:** Standardized effects between variables (β).

**Path**	**Direct effect**	**Indirect effect**	**Total effect**
RC–>RP	−0.095	0.000	−0.095
RC–>NE	0.000	−0.029	−0.029
RC–>PCB	0.141	−0.003	0.139
RC–>ECB	0.000	−0.007	−0.007
DT–>RP	−0.219	0.000	−0.219
DT–>NE	0.000	−0.068	−0.068
DT–>PCB	0.482	−0.006	0.476
DT–>ECB	−0.118	−0.016	−0.134
RP–>NE	0.309	0.000	0.309
RP–>PCB	0.000	0.027	0.027
RP–>ECB	0.000	0.073	0.073
NE–>PCB	0.088	0.000	0.088
NE–>ECB	0.235	0.000	0.235

*RC, risk communication; DT, degree of trust; RP, risk perception; NE, negative emotions; PCB, protective coping behavior; ECB, excessive coping behavior*.

## Discussion

The results showed that the nine direct paths of the model hypothesis, except for the path between RC and ECB, reached significance. The results of the model analysis are discussed below according to the standardized path coefficients and load coefficients of each component.

Government regulation of the public's RP based on the current pandemic situation is a key part of emergency risk management. Our results showed that RC and DT had a direct negative effect on RP, indicating that those responsible for risk management need to use relevant media to convey appropriate guidance to inform public opinion; promote RC; and strengthen the public's DT in self-protection, medical workers, and the government, thereby more effectively regulating the public's RP. The spread of pandemic information can significantly affect the public's RP, and the media largely construct the public's perception of risk. In the early stages of prevention and control of the pandemic, people used various media to obtain relevant information and to understand the development of the pandemic and how society in general was responding. The results showed that the largest source of public information or knowledge about COVID-19 was the Internet (80.3%), which may be partly related to the increase in internet use during the pandemic. Research has shown that 46.8% of 6,416 Chinese people increased their dependence on the Internet and 16.6% had longer hours of internet use during the COVID-19 pandemic (51). However, as the public began to focus attention on the pandemic, media-related information became complex and extensive and often contained rumors mixed in with more factual reporting. The pandemic has accelerated an already-growing trend in users' interest in fake news sources (52). Therefore, it is necessary to disseminate scientific information in real time to respond to public health emergencies, and rapid sharing of scientific information is an effective way to reduce public panic about public health emergencies. The information media mainly comprised official media, such as government documents and press conferences (70.7%) and social networks (69.4%), indicating that emergency management authorities should pay attention to the information transmission function of official channels and new mass media. Research has shown that unofficial social media causes urban citizens to experience higher levels of panic than official media (53). The government's posting and media posting can significantly contribute to a more informed public perception of risk issues; therefore, the government and media must be vigilant in providing credible risk-related information (54).

Faced with a pandemic, PCB will substantially influence the effectiveness of risk control measures performed by the government (19, 20); thus, identifying the factors affecting public behavior is critical to emergency management. Our results showed that RC and DT had direct and indirect effects on public behavioral coping strategies during the COVID-19 epidemic. Our results were consistent with the previous literature (12, 13, 17). RC directly and positively affected the public's PCB; the higher the degree of RC, the more likely the public were to adopt PCB. However, the results did not show that RC had a direct effect on ECB, indicating that PCB and ECB undertaken by the public were not mutually exclusive as both could be engaged in simultaneously.

The results showed that DT in self-protection capabilities, medical workers, and the government's ability in relation to effective control of the pandemic had a direct positive effect on PCB and a negative effect on ECB, suggesting that building social trust is a key measure for promoting effective public awareness in terms of epidemic prevention.

Public behavior was also driven by intrinsic psychological factors (34). An online survey in the Italian population indicated that the COVID-19 pandemic may be an influential risk factor for the development of psychological diseases (35). The COVID-19 pandemic could be considered as a traumatic event (37), which had clearly affected the mental health of the public, particularly closely linked to negative emotions (39), sleep disorder (36), and PTSD (37). However, previous studies have mostly focused on the effect of influencing factors, such as RC and trust on public behavior, the psychological mechanisms and relationships involved have rarely been discussed. In our study, we not only focused on the direct effects of influencing factors on public behavior, but also on the indirect effects of psychological factors. On the one hand, external influencing factors, such as RC and trust, could influence public psychological factors, such as public RP and emotion, which in turn could affect public behavior. However, the indirect effect appears to be relatively small. On the other hand, RP can positively affect public behavior, possibly through the mediating effect of NE. Interestingly, with the increase in RP, NE also grew, which had the effect of not only promoting public PCB but also of promoting ECB, indicating that in risk control, attention should be paid to public emotional stability and to avoiding too high or too low RP.

In conclusion, our hypothesis was verified. We used an SEM to evaluate the internal potential relationships between influencing factors and coping behaviors of the public facing COVID-19 risk. Compared with conventional statistical methods, the analysis results of the SEM were likely to be more comprehensive as the theoretical model constructed in this study had an excellent fit, as indicated by the various fit indices. The results showed that RC, DT, RP, and NE were significantly directly or indirectly related to public behavior. The reported findings provide useful information for emergency risk management and a theoretical basis for follow-up research on public coping behavior during a pandemic. Furthermore, the research results on the relevant formation mechanisms involved and the relationship between factors and behaviors are likely to be useful in guiding subsequent research.

However, this study had several limitations. First, due to the pandemic, the questionnaire was applied using convenience sampling on the Internet so that only certain types of respondents may have become involved. Specifically, the respondents were all internet users, were often relatively young, and could not be considered representative of the entire population. However, considering the high internet penetration rate in China (71.6%) and that the proportion of mobile internet users was reported to be 99.6% (as of June 2021) (55), we consider that this limitation is unlikely to have led to excessive distortion. Second, our theory (Figure 3) is verified by investigation only in China and limited in scope due to its focus on mainly young people. However, considering that the basis of our hypothesis come from the investigation and research of different countries as seen in Introduction (5, 6, 15, 25, 35), our study could be extended to accommodate a wider range of applications and provides more opportunities for further investigation. Third, although our questionnaire was self-designed based on the latest relevant literature, it inevitably contained some biases. In this study, we explored a public behavior model from the perspective of psychological factors only. Further studies should be conducted to improve the current questionnaire design to better capture other potential factors. For example, according to the relevant literature (4, 35, 36), we could speculate that factors like sleep quality may have the mediating effect in psychological behavior model during COVID-19 pandemic. Besides, additional considerations could be included, such as personal characteristics, health literacy, social environment, and other aspects (5–7), in ongoing model research for the public behavior model designed further optimized in future.

## Data Availability Statement

The original contributions presented in the study are included in the article/supplementary material, further inquiries can be directed to the corresponding author/s.

## Ethics Statement

The studies involving human participants were reviewed and approved by Gannan Medical University. Written informed consent to participate in this study was provided by the participants' legal guardian/next of kin.

## Author Contributions

JG and RH conceived this study and designed the questionnaire. JG performed data analysis, interpreted the results, and wrote the paper. RH, XW, JT, and WY collected the relevant data. CW contributed to the interpretation and revision of the paper. All authors have read and approved the final manuscript.

## Funding

This work was supported by Humanities and Social Sciences Project of Jiangxi Colleges and Universities (No. JC21204) and the Social Science project of Ganzhou (No. 2021-018-0008).

## Conflict of Interest

The authors declare that the research was conducted in the absence of any commercial or financial relationships that could be construed as a potential conflict of interest.

## Publisher's Note

All claims expressed in this article are solely those of the authors and do not necessarily represent those of their affiliated organizations, or those of the publisher, the editors and the reviewers. Any product that may be evaluated in this article, or claim that may be made by its manufacturer, is not guaranteed or endorsed by the publisher.
